# Someone is to blame: the impact of suicide on the mind of the bereaved (including clinicians)

**DOI:** 10.1192/bjb.2024.37

**Published:** 2025-02

**Authors:** Rachel Gibbons

**Affiliations:** Royal College of Psychiatrists, London, UK

**Keywords:** Suicide, suicide bereavement, psychodynamics, suicide loss, trauma and stressor-related disorders

## Abstract

This paper presents an emerging understanding of the psychodynamics of suicide loss, derived from over 1500 accounts of suicide bereavement shared by families, friends and clinicians. It identifies clear patterns in the responses of the bereaved, particularly the formation of delusional narratives that often place them at the centre of blame for the death. These narratives have a profound impact on well-being, increase the risk of mental illness and elevate the likelihood of death by suicide. They not only cause harm to the bereaved but also permeate and distort our systemic and societal responses. Understanding why suicide unleashes such painful and dangerous forces helps mitigate the widespread harm and distress that often follows such a death. This knowledge also enables us to effectively and compassionately support those bereaved.

In my previous paper, ‘Eight “truths” about suicide’, I described my experience in the first 3 months of being a consultant psychiatrist, when three patients who had been under my care died by suicide.^1^ The impact of these deaths shattered the picture I had of the clinician I would be and my understanding of the nature of the psychiatric work I had been longing to do. This profound and painful experience defined my personal and professional life from that point on.

Over the past 15 years I have heard around 1500 cases of suicide bereavement from families, friends and clinicians. Clear patterns have emerged from these accounts, both in the nature of suicide itself and in the response of those bereaved. In the previous paper I explored the nature of suicide. In this companion piece, I intend to share the emerging understanding of the psychodynamics of suicide loss. By apprehending why suicide unleashes such painful and dangerous forces we can mitigate the widespread harm and distress that often follows a death of this nature and encourage and support the possibility of psychological growth.^2^

Suicide bereavement is exceedingly common. In various settings I have observed, in keeping with the research, that approximately 60–70% of individuals have been affected by suicide in some aspect of their lives at least once, with many encountering it multiple times. This exposure has widespread consequences. It profoundly affects well-being, increases the risk of mental illness and elevates the likelihood of death by suicide.^3–8^ The deaths that I experienced had a profound impact on my mental health. The moment that I was told about the first patient who had died, I suffered a catastrophic psychological shock, invisible to others, that fractured my view of the world and my place in it. Prior to this I believed that I would know who was at risk of suicide and could intervene to prevent it. In retrospect I know now that this was an oversimplified and omnipotent belief that was supported and encouraged by the systems around me. At the time, I underestimated the magnitude of the destructive forces inherent in human nature, as well as the challenge posed by psychiatric work, where confronting these forces is a daily reality.

Following these deaths, I developed a clear conviction that I was to blame. I felt this so strongly that I did not confide my fears to anyone, believing that they would be shocked and agree with me that these deaths were my fault. Looking back, what amazes me is that I continued to work while being at the epicentre of this post-traumatic emotional storm. This is a testament to my ability to divide (split*) my personality (ego*) into ‘work’ and ‘non-work’ parts, a trait I share with many other doctors (terms marked with an asterisk are defined in Table 1). What did help me at this time was getting together with colleagues, similarly affected, in a peer support group designed specifically to process the effect of the death of a patient by suicide. This group is still running after 15 years and has modelled the development of other similar groups in different environments.

**Table 1:** Definitions of terms

Term	Definition
Split in the ego	A division in the sense of self, where two parts of the self function side by side without influencing each other, one taking reality into account and the other disavowing reality. Each side not communicating with the other.
Ego	One of three parts of Freud's ‘structural model’ of the psyche that mediates between the ‘id’ and the ‘superego’. The ego engages with reality and it is where self-identity is located. The id is entirely unconscious and holds the basic instinctual urges, such as aggression and sexuality. The superego functions as a conscience, repressing what it considers to be morally unacceptable.
Primary ambivalence	In ambivalence there are two contrary wishes at the same time. In someone who is suicidal it is the wish to live and the wish to die. In a primary ambivalent state these wishes are held by two different parts of the individual owing to the split in the ego.
Psychic defences	Socially unacceptable ideas, anxiety-producing wishes or desires, traumatic memories and painful emotions are held outside of conscious awareness by unconscious defence mechanisms. These take many different forms.
Projection	An unconscious defence to expel unacceptable aspects of the self into the outside world and then experiencing them as belonging to someone or something else.
Acting out	Engaging in actions that convey certain feelings to others rather than consciously acknowledging them oneself. The first sign of feeling one way is through acting that way.
Mentalise	The ability to reflect on and to understand your own state of mind. To have insight into what you are feeling and why. To be able to conceptualise other people's mental states and to recognise that they may be different from your own.
Symbolise	A symbol is an indirect form of representation that allows an individual to think about people, objects and events that are not concretely available. These symbols are available for utilisation intrapsychically to represent ideas, conflicts or wishes.
Reaction formation	Changing an unacceptable feeling into its polar opposite to make it less threatening – for example, love into hate or insecurity into bravado.

The case examples in this paper are composites of cases taken from clinical practice, those recounted by relatives and other survivors of suicide, and data from many sources, including audits in mental health organisations, the police and transport services, and coroners’ records.

## The psychodynamics of suicide loss: the development of a delusional narrative

*While it might seem obvious that the individual who killed themselves should be held responsible, in my clinical experience, this only happens some of the time, and for only some of the survivors of a suicide loss. Instead, most survivors begin by blaming themselves for the death. [ … ] Sometimes, survivors will also assign the blame to someone else, such as other family members, friends, or professionals [ … ] In my experience, it is less common for survivors to begin by blaming the deceased, although that may come in.*Jordan*^9^*

The loss of someone close to us by suicide profoundly affects our psyche, rendering the mourning process more complex and challenging.^10^ The impact results from different and unique aspects of the death:

- Suicide itself is a shocking annihilatory loss; someone is suddenly irretrievably gone, in most cases with no clear warning.^1^
- Those bereaved face unresolvable uncertainty about how something so tragic has occurred.^1^
- Suicide takes place in a state of primary ambivalence*, characterised by a split in the ego. This means that there is a part of the individual that wants to live (the victim) and another part that is split off, planning the death (the perpetrator). The two parts do not interconnect and appear to function independently. This can lead to confusing messaging preceding the death, depending on which part is communicating. Those who are bereaved have often been reassured and distracted by the ‘perpetrator’ part before the death, leaving them with profound guilt and bewilderment in the aftermath.^11^
- In the period immediately after the death, there is a need to preserve the memory of the person who has died. To do this they have to remain the ‘victim’ and the ‘perpetrator’ needs to be found elsewhere. This results in a profound unrecognised ambivalence in the bereaved, who find themselves torn between continuing to love the person who is gone and hating them for their actions. This hatred initially has to be repressed to maintain the idealisation of their lost loved one and the mourning process stalls. This leads to a state of complex bereavement, accompanied by a high risk of clinical depression.^10,12^
- Suicide is a relational event. Those who are bereaved can receive devastating communication through projections* from the person who has died, evacuated through the ‘acting out’* of the act itself. These are painful pieces of the internal world of the deceased. These projections cannot be returned and are very hard to bear.^1,11^
- Suicide exposes the bereaved to the disturbing reality of the destructive forces that underly all humanity, and that the seeds of suicide may be sown in every human heart.
- The profound stigma still associated with suicide loss leads to an immediate sense of shame that inhibits grieving.

Overall, suicide has been likened to a ‘cluster bomb’ (Irma Brenman Pick, psychoanalyst), with its multiple impacts shattering our fragile construct of life, often referred to as our ‘assumptive world’.^13,14^ Consequently, our mind undergoes a temporary fragmentation.^15,16^ This mental disintegration is unbearable and as narrative beings, we resolve this by crafting stories to alleviate the pain and uncertainty. Even a fabricated story is preferable to the anguish of a fractured psyche. So after suicide, we create a narrative, embraced with delusional intensity, to soothe our shattered minds and to preserve the memory of the deceased. However, this temporary reprieve comes at a cost, as the mind retains an encapsulated delusional belief that impedes the grieving process. It is important to note that delusional and psychotic processes are not exclusively symptoms of illness but also components of normal human mental functioning. Parts of the mind, secretly engaged in psychotic functioning, can come to the fore when subjected to a loss that threatens to overwhelm the ego.^17,18^

In the following quote from Freud, he is advancing the understanding of delusions, suggesting that they are not the problem themselves, but rather a common and human solution to a damaged ego:

*The delusion is found applied like a patch over the place where originally a rent had appeared in the ego's relation to the external world* (p. 215).^19^

### The structure of the delusional narrative

These delusional narratives are quite straightforward and follow a similar pattern. In these stories, the bereaved individual is the central figure (the protagonist), who believes they have made a fatal error, a sin of omission or commission, leading to the death. These stories can seem plausible, making them a challenge to identify. For example ‘I didn't answer the phone when he called me; I was just tired of it’, or ‘I didn't prescribe an antidepressant in the last ward round when he asked me to’ or ‘I did prescribe him the antidepressant in the last ward round when he asked me to’. They can also be more clearly bizarre. I believed that the first young man who died looked at me at our last meeting and without words conveyed his intention to die by suicide, which I ignored. Now, I can identify this as a delusional narrative, which reduces its power over me, but I do still believe it.

*Case example: X was certain that D had sent her a special message before he died to warn her of his intention. She was clear it was either a picture or a letter. She believed that if she had found it, she could have prevented his death. She spent weeks searching all over the building, in drawers, cupboards and asking people about its whereabouts. However, there was no evidence that any communication of this nature ever existed*.

These delusional narratives start to take shape swiftly following the shock of learning about the death, often within approximately 20 min.

*Case example: Dr P was told that a patient she knew well had died in a manner that looked like suicide. Dr P was profoundly shocked, turning white as a sheet and repeatedly muttering ‘I am shocked, I did not expect this to happen, he was the last person I thought would die like this’. However, within 20 min, she began constructing a narrative in which she believed she had missed clear signs of his intentions during their last meeting, blaming herself for not recognising them for what they were. This narrative gained momentum over the following days*.

However logical these narratives may seem, they are hypothetical and unverifiable. Creating simple stories can be helpful for us in the early stages of grief, relieving unbearable uncertainty, minimising the role of the deceased in their own death and protecting them from our anger at their actions.

If grief is tolerated and mourning is supported, these delusional narratives can lose some of their power. Gradually, over time there can be a shift in how we preserve the memory of the deceased, moving beyond viewing them solely as a victim. This opens the door to a more nuanced understanding, allowing for the exploration of questions about agency and responsibility:

*Now, I don't think I will ever find the answer to the question that has plagued me and many who suffer the loss of a loved one to suicide. That question is ‘Why?’. We even have the question of who, because when my son died, he became somewhat unrecognisable. Who was he? Who was the agent that had caused this death? I couldn't contemplate the idea that it was really him. So, I was obviously looking for other causes of his death, something that would enable me to continue my relationship with him and my love for him. It must have been something in his brain, something in his chemistry, some external agency, or maybe the failure of a health care system. Something else, because to actually attribute it to him was an almost intolerable thing. Then, almost immediately, we start telling ourselves those stories – all the things I could have done and should have done. That searing guilt surrounds us. *Professor David Mosse, Chair of the Haringey Suicide Prevention Group and a bereaved father**

### Rethinking the nature of ‘blame’ in the context of grief

When the mind suffers the impact that follows a death by suicide, it is overwhelmed by anxiety, and the capacity for mentalisation*^20^ (the ability to understand and interpret mental states) and symbolism* is often lost. In this state, where abstract thinking is impaired, there is a tendency to think concretely, with an inability to grasp nuances. ‘Blame’ arises from this state of mind and becomes integrated into these delusional narratives. It implies a singular known cause, oversimplifying the complex, multifactorial and ultimately unknowable nature of suicide. ‘Responsibility’ is a concept from a mentalising perspective, acknowledging multiple contributing factors. This allows us to consider our own role and explore various scenarios, including the part played by the deceased in their own demise. If anyone uses the word blame after a death by suicide then it indicates that they have lost their capacity to mentalise and need help and support to recover their capacity to reflect.

## The risks of entrenched delusional narratives

*Suicide freezes the relationship in the zenith of its sadism*.Campbell and Hale^11^

### To those bereaved

Understanding the strength and nature of these delusional narratives is crucial in understanding the dangers posed by suicide bereavement. Believing oneself to be ‘to blame’ for a loved one's death can be unbearable. This belief is often compounded by intense projections from the deceased, which may include unconscious accusations of ‘blame’. Understandably, this can severely affect the mental health of the bereaved. In some cases, it may overwhelm the survival instinct, potentially leading to death by suicide as a means of escape.^6,7,21^ This phenomenon is considered a mechanism in the transmissibility of suicide within clusters.^22^ These delusional narratives also are held onto in an obsessional way, blocking the mourning process and inhibiting the development of those bereaved, who can see recovery and re-engagement with life as a betrayal of their loved one:

*the dead person is the apparent victim, but the true victim is the one that stays alive, for he or she has to live with what they feel they might have caused [ … ] the survivor directs enormous resistances to any change or working through of this state, almost as a memorial to the dead* (p. 41).^11^

### To those surrounding the bereaved

*The institution, as with any group, will find its vulnerability exposed and unconscious retributive sadism may be excited and scapegoating ensue in order to get rid of the institutional responsibility. Inevitably this will lead to splits in the functioning of the organisation. Thus clinicians, already overwhelmed by guilt and internally persecuted, now feel accused and on trial in front of their own organisation. The internal ruminations over a perceived ‘fatal mistake’ may now result in a masochistic presentation to the panel and the possibility of volunteering for being the scapegoat*.Campbell and Hale^11^

Our strong belief in these narratives frequently results in the tendency to assign blame to others for our own self-preservation. We do this by using the psychic defences* of projection* and reaction formation*. Given the good evidence that there is an increased risk of suicide in those bereaved, to exonerate ourselves by blaming others, such as a family member, a clinician or mental health services, for a period of time after a death may reduce the risk of re-enactment. The bereaved can then proactively seek, mobilise and strive for change and justice, restoring a sense of meaningfulness and self-worth. Although this can drive positive changes in structures and services, if not understood, it can also lead to negative outcomes such as uncontrolled scapegoating where ‘someone needs to be held accountable’. It is possible that factors such as mental healthcare could have played a role in the outcome, but this remains entirely speculative and fear of blame and scapegoating can hinder open-hearted engagement with those in a suicidal state of mind.

### To our systems and society

These delusional beliefs can permeate all our systems and processes, dominating the discourse and influencing cultural belief, future developments and societal actions. In the past month, I have read narratives in the media following a death by suicide that blame schools, hospitals, ‘bullies’, COVID-19, social media and various individuals who are named and shamed. Our complicity and inability to challenge these narratives reveal much about the primitive aspects of human nature.

One such delusional narrative that has permeated the mental health sector is the belief that risk assessment tools predict the likelihood of an individual dying by suicide in the future. Even given clear evidence and national guidance to the contrary this practice continues to be widespread, influencing service development and provision and dominating clinician–patient interaction.^23,24^ Another related narrative is that failure to complete these risk assessment tools contributes to deaths, frequently being identified as a failure and learning point in organisational serious incident reviews and coroners’ reports.^25^

The processes following a death and any attempts to ‘learn’ from it can be profoundly distorted owing to the destructive forces unleashed within organisations, which may react protectively as if they were a sentient entity, seeking a scapegoat to preserve their own integrity and reputation. Clinicians affected by the death by suicide of their patient and burdened with a delusional narrative of guilt can unconsciously assume this role of the scapegoat. It is not uncommon for staff members to be suspended in the immediate aftermath of a patient's suicide, a practice that should be regarded as scapegoating unless unequivocally proven otherwise. This can also have an impact on and interfere with clinicians giving evidence in the coroner's court, where feelings of guilt may lead them to metaphorically raise their hand to accept projections of blame, distorting the coronial process.

## Factors increasing the impact on those bereaved

Beyond the profound direct impact of a death by suicide on the bereaved, research suggests that additional factors in the circumstances surrounding the death can further intensify its effect on those grieving. Understanding these factors facilitates a more comprehensive insight and allows for the establishment of measures to lessen their impact. These factors include:

- Finding the body: this adds a serious further layer of trauma.^26^
- Notification about the death: when handled insensitively this magnifies the initial emotional effects, negatively influences grief responses and increases the risk of mental disorder.^24^
- Early assumptions about the death, including the possibility of homicide, can distort the response.
- Disenfranchised grief: this refers to the perception that there is not enough grief to go around and that some groups or individuals have more right to grieve than others. Clinicians are particularly affected, feeling that their grief will compete and detract from the family's grief and pain.^27^
- Organisational and systemic response: unsupportive responses increase the traumatic impact and inhibit recovery, whereas effective compassionate support mitigates the damaging personal and professional effects and facilitates post-traumatic growth.^27–29^

## Conclusion

*My journey has gone from private grief and bewilderment and utter confusion, to sitting in forums and different kinds of public spaces talking about suicide in a community, as a collective, as a kind of public concern, a national concern [ … ] these changes are all bound up with a journey of grief and living with this tragedy [ … ] It's changed everything. *Professor David Mosse, Chair of the Haringey Suicide Prevention Group and a bereaved father**

The best outcome in suicide bereavement is a terrible transformational journey and at worst it can be deadly. Understanding the psychodynamics can aid in reducing the likelihood of harm and enables us to provide meaningful support. The delusional narratives that emerge following a death obstruct the mourning process and torment those bereaved. Recognising the need for these narratives means that we can resist their pull, encourage their exposure and meet them with compassionate and gentle challenge. This profoundly helps those bereaved, unhooking them from masochistic suffering after a death. If successful, the mourning process can then proceed with less resistance.

There is a pressing need for a collective opposition to these delusional narratives: to assert emphatically and unequivocally that ‘no one is to blame for anyone else's death by suicide’. However, to achieve this we must begin with ourselves. We need to challenge our own unyielding self-accusations after bereavement from suicide. Only after tackling this personal challenge can we effectively address the issue across our systems, as well as more broadly in society and the media.

For new RCPsych guidelines for all mental health organisations on the pastoral care of their staff following a death by suicide of a patient please see College Report CR234.^30^

## Data Availability

Data availability is not applicable to this article as no new data were created or analysed in this study.

## About the author

**Rachel Gibbons** is a consultant psychiatrist, psychoanalyst and group analyst. She works independently. She is chair of the Working Group on the Effect of Suicide and Homicide on Clinicians and the vice-chair of the Faculty of Medical Psychotherapy at the Royal College of Psychiatrists, London, UK.
